# *DnFCA* Isoforms Cooperatively Regulate Temperature-Related Flowering in *Dendrobium nobile*

**DOI:** 10.3390/biology12020331

**Published:** 2023-02-19

**Authors:** Ting Pan, Ning-Meng Deng, Wu-Xia Guo, Min-Zhen Wan, Yan-Tong Zheng, Song-Yi Chen, Chuan-Liang Liu, Hong-Bo Li, Shan Liang

**Affiliations:** 1 Guangdong Provincial Key Laboratory of Biotechnology for Plant Development, School of Life Sciences, South China Normal University, Guangzhou 510631, China; 2 School of Bioengineering, Zhuhai Campus, Zunyi Medical University, Zhuhai 519041, China

**Keywords:** orchid, *Dendrobium*, FCA, temperature, flowering, 3′ alternative polyadenylation

## Abstract

**Simple Summary:**

A low temperature is required for flowering in *Dendrobium nobile*. However, this process can be suppressed or disrupted by a high ambient temperature. Little is currently known about the regulation networks and the mechanisms behind this process. Here, we report two isoforms from the *DnFCA (FLOWERING CONTROL LOCUS C in D. nobile)* gene locus, *DnFCAγ* and *DnFCAβ*, which cooperatively regulate temperature-related flowering in *D. nobile*. The overexpression in *Arabidopsis* indicated that both isoforms can partially rescue the late flowering of *fca*-*1* but tend to delay the flowering time and downregulate the *APETALA1* (*AP1)* expression in wild-type plants. When introduced into the detached axillary buds and seedlings of *D. nobile*, only *DnFCAγ* was able to suppress the transcription of *DnAPL1* (*AP1-LIKE 1 in D. nobile*) in axillary buds, but both of them activated *DnAGL19 (AGAMOUS LIKE 19 in D. nobile)* in seedlings. Vernalization induced the accumulation of *DnFCAβ* in leaves, which allowed the activation of *DnAGL19* and *DnFT* for the initiation of the inflorescence meristem (IM) in axillary buds. The synchronous enrichment of *DnFCAγ* in axillary buds may result in the suppression of *DnAPL1*, avoiding the premature development of floral organs until the floral primordium is produced after a long period of low temperatures. A high ambient temperature induced the long-standing accumulation of *DnFCAγ* in axillary buds, which can lead to the loss of flowering competence due to the lack of *DnAPL1* activation.

**Abstract:**

Timely flowering is a determinative trait for many economically valuable species in the *Dendrobium* genus of the Orchidaceae family, some of which are used for ornamental and medicinal purposes. *D. nobile*, a representative species of nobile-type *Dendrobium*, normally flowers in spring after exposure to sufficient low temperatures in winter. However, flowering can be stopped or disrupted by the untimely application of high temperatures. Little is known about the regulation and the mechanisms behind this switch. In this study, we report two isoforms from the KFK09_017173 locus of the *D. nobile* genome, named *DnFCAγ* and *DnFCAβ*, respectively, that cooperatively regulate flowering in *D. nobile*. These two isoforms are generated by alternative 3′ polyadenylation of *DnFCA (FLOWERING CONTROL LOCUS C in D. nobile)* pre-mRNA and contain a distinct 3′-terminus. Both can partially rescue late flowering in the *Arabidopsis fca*-*1* mutant, while in wild-type *Arabidopsis*, they tend to delay the flowering time. When introduced into the detached axillary buds or young seedlings of *D. nobile*, both were able to induce the transcription of *DnAGL19* (*AGAMOUS LIKE 19 in D. nobile)* in seedlings, whereas only *DnFCAγ* was able to suppress the transcription of *DnAPL1 (AP1-LIKE 1 in D. nobile)* in axillary buds. Furthermore, the time-course change of *DnFCAγ* accumulation was opposite to that of *DnAPL1* in axillary buds, which was remarkable under low temperatures and within a short time after the application of high temperatures, supporting the suggestion that the expression of *DnAPL1* can be inhibited by a high accumulation of *DnFCAγ* in floral buds. In leaves, the accumulation of *DnFCAβ* was in accordance with that of *DnAGL19* and *DnFT* (*FLOWERING LOCUS T in D. nobile)* to a large extent, suggesting the activation of the *DnAGL19–DnFT* pathway by *DnFCAβ*. Taken together, these results suggest that the DnFCAγ–DnAPL1 pathway in axillary buds and the DnFCAβ–DnAGL19 pathway in the leaves cooperatively promote flowering under low temperatures. The long-term and constant, or untimely, application of high temperatures leads to the constitutive suppression of *DnAPL1* by a high level of *DnFCAγ* in axillary buds, which consequently delays floral development.

## 1. Introduction

Many species belonging to the *Dendrobium* genus of the orchid (Orchidaceae) family are economically valuable and can be used for ornamental or medicinal purposes. There are two classes of *Dendrobium* species, represented by *D. nobile* and *D. phalaenopsis*, respectively, that are evolutionarily adaptive to low or high ambient temperatures and bloom in different seasons. The nobile-type *Dendrobium*, such as *D. nobile*, *D. catenatum*, and *D. chrysotoxum*, require sufficient low temperatures in winter (namely, vernalization) for flowering in spring [1,2,3,4,5,6]. However, the extent of the low temperatures and the duration required varies depending on the species or hybrid cultivars, significantly affecting the flowering time, flower number per inflorescence, and the size and longevity of a single flower in these species [1,4,5,6]. The untimely application of low or warm/high temperatures will result in abnormal flowering phenotypes, such as early/delayed flowering, non-flowering, and floral reversion. As early as the 1950s, Rotor concluded that a constant temperature of 18 °C suppresses flowering in *D. nobile* [2], which may be due to the lack of vernalization. High temperature is also able to lead to the outgrowth of offshoots (also known as “Keiki”) at nodes along the stalks, as previously described by Kosugi [3]. Offshoots are common under non-optimized environments, including, but not limited to, high temperatures. This is a benefit for *Dendrobium* plants and may increase the propagation potential to overcome certain environmental predicaments. However, the generation of offshoots is not always associated with a non-flowering phenotype, although the inflorescence and offshoot both compete for the same nodes. In a word, vernalization is obligatory for nobile-type *Dendrobium* to initiate flowering, while an elevated temperature has dual effects of stopping flowering and promoting offshoot outgrowth. Unfortunately, the underlying mechanisms are, as yet, largely unclear.

To initiate flowering, plants must pause vegetative growth before the initiation of reproductive growth, in a process called phase transition. In *Arabidopsis*, there are six known pathways for the convergence of signals of developmental and environmental cues on flowering integrators, including FLOWERING LOCUS C (FLC), FLOWERING LOCUS T (FT), SUPPRESSOR OF CONSTANS (SOC1), and LEAFY (LFY), ultimately leading to inflorescence meristem (IM) determination by AGAMOUS LIKE 24 (AGL24) and LFY at the shoot apices [7,8,9]. Subsequently, the *APETALA1* (*AP1*) gene is activated, resulting in the activation of the floral homeotic genes to specify floral organs [10,11]. To commit to floral development, the balance between the determinants for IM and those for the floral meristem (FM) must be finely tuned [11,12]. AP1 is involved in the central hub and is required for IM-to-FM transition and for the identity of sepals and petals [13]. *AP1* expression, observed throughout the young floral primordia at the earlier stages of floral development and which is timely and spatially regulated to ensure the subsequent specification of the outer whorls of the floral buds [13,14], can serve as an indicator of the initiation of flower development. In agreement with this idea, the loss-of-function mutant of *AP1* (e.g., *ap1*-*1*) presents phenotypes of floral reversion, in which FM is switched from a determinate back to an indeterminate status [11,13,15]. The expression of *AP1* and its paralogous gene *CAL* can be induced by LFY and FD (AtbZIP14) (in concert with FT) at IM, while the AP1 protein can, in turn, give feedback to the expression of *LFY.* This positive loop allows the continued activation of *AP1* [16,17,18]. At the same time, the expression of *TERMINAL FLOWER 1* (*TFL1)* is repressed by LFY, AP1, and CAULIFLOWER (CAL), preventing the premature termination of the floral transition [15,18,19]. On the other hand, genetic interaction has revealed that AP1 can inhibit the action of *FCA*, which is an upstream activator of *FT* and *LFY* [20], suggesting a negative loop between AP1 and the flowering-time genes. This implies that AP1 contributes to the ending of the floral transition to some extent. The molecular functions and regulatory networks of *AP1* homologs in monocotyledonous plants are different from those in *Arabidopsis* [14,16,21]. For example, *VERNALIZATION1* (*VRN1)*, an *AP1*-like gene in wheat, acts in the vegetative-to-reproductive phase transition and can be induced by vernalization [21,22,23]. *VRN1* and *FT1* mutually activate each other, forming a positive feedback regulation loop in response to low temperatures and photoperiods [24,25]. This loop may be disrupted by VERNALIZATION1 (VRN2), which is activated in the fall season and downregulates *VRN1*, resulting in the silencing of *FT1* and flowering suppression [24].

FLOWERING CONTROL LOCUS C (FCA) is considered a regulator of flowering and acts upstream of *AP1* through the autonomous pathway and the vernalization pathway [20,26]. The *FCA* gene was primarily identified in *Arabidopsis* but subsequently also found in rice, barley, and other species [26,27,28,29]. The alternative 3′ polyadenylation (3′ APA) of *FCA* pre-mRNA occurs frequently and is conserved across species, producing a conserved *γ* isoform and a very different *β* isoform [27,29]. The *Arabidopsis FCAγ* isoform has been extensively studied; it is produced by 3′ polyadenylation at the distal canonical polyadenylation site (PAS) and contains a full-length open reading frame (ORF) for the functional protein [26]. The deduced FCAγ protein may promote floral transition via diverse mechanisms. For example, it promotes the maturation of miR172 under warm conditions, which ultimately leads to the miRNA-guided degradation or translation suppression of the members of the *AP2*-like subfamily and results in the activation of *FT* expression [30,31,32]. FCAγ also promotes the production of non-coding RNA, *COOLAIR*, which may inhibit the biosynthesis of *FLC* mRNA in response to vernalization [33,34]. Signals from these pathways will subsequently converge to flowering integrator genes such as *SOC1* and *LFY*, ensuring the activation of *AP1* and flowering under warm temperature. The *β* isoform is not involved in flowering regulation in *Arabidopsis* and is considered a balancer of *γ* isoform yield [26].

Orthologs of *AP1* have been identified from various orchid species, and some have been functionally characterized [35,36,37,38,39]. We previously identified the *DnAPL1* gene (also named *DnVRN1* in [40]) from *D. nobile*, which codes for an AP1/FUL-like MADS-box protein [41]. Similar to *AP1* homologs from *Arabidopsis* and wheat, the overexpression of *DnAPL1* can accelerate flowering and alter the flower patterning in *Arabidopsis*, indicating that DnAPL1 is conserved in floral transition and in the determination of FM identity [11,21,41]. During the development of floral buds, the expression of *DnAPL1* is weak in Stage 1 and 2 floral-AXBs but is dramatically increased after Stage 3 [41]. However, the regulation of *DnAPL1* expression remains unclear. In this study, we report two isoforms of the *DnFCA* gene locus. The *DnFCAγ* isoform may act as a repressor of *DnAPL1* in AXBs, while the *DnFCAβ* isoform may work in the leaves, where it acts as an activator of *DnAGL19*. The contributions of these regulations to flower development under different temperatures are discussed.

## 2. Materials and Methods

### 2.1. Plant Materials and Growing Conditions

*D. nobile* plants were pot-cultured in the garden on the campus of South China Normal University (SCNU) under natural temperatures and day length until treatment. To create transgenic *D. nobile*, detached Stage 0 (untreated) AXBs of adult stalks were freshly collected from Oct. to Dec. every year; young seedlings were generated by explant culture using seeds as starting materials. For the temperature treatment, *D. nobile* plants containing 3-year-old adult stalks were treated at 27 °C or 15 °C in culture chambers, and then AXBs were collected from these stalks after the indicated number of days. For the 15 °C treatment, all AXBs collected within 50 days were at Stage 1, and as the treating time extended, the AXBs became more developed and some Stage 2 AXBs were included. For the 27 °C treatment, the AXBs at each time point were at Stage 1. The developmental stage of each AXB was identified as described in [41]. The samples for spatio-temporal expression detection were collected from 3-year-old adult stalks of nonvernalized plants.

### 2.2. Isolation of Alternative Polyadenylation Isoforms

The 3′ RACE technique was used to isolate isoforms generated in alternative 3′ polyadenylation. The total RNA was extracted from the AXBs of *D. nobile* using the CTAB method, as described previously [40,42]. Then, 1 μg total RNA was reverse transcribed (RT) using MMLV reverse transcriptase (TOYOBO, Japan) and the anchor Oligo (dT) primer (Table S1) in a final volume of 20 μL, generating first-strand cDNA that contained an adaptor sequence at the 5′ end and could be bound by 3′ PCR primer (Table S1). A specific forward primer, DnFCA-γ-F (Table S1), matching the upstream region of the starting codon “ATG” was designed and paired with the 3′PCR primer in the later PCR reactions, aiming at the amplification of the full-length open reading frame (ORF) together with the 3′UTR fragment of each *DnFCA* 3′ APA isoform. Then, 1 μL RT products were used as templates in the PCR reaction and were amplified using B-Taq plus DNA polymerase (TOYOBO, Japan). The resultant amplicons were recovered and sequenced (Sangon Biotech, Shanghai, China), followed by BLASTN analysis against the reference genome. The primers are listed in Table S1.

### 2.3. Generation of Arabidopsis Transgenic Lines and Phenotype Analysis

To discover the biological functions of the *DnFCA* isoforms, the full-length ORFs of the *γ* and *β* isoforms were amplified using specific primers (Table S1), followed by fusion with the 35S promoter of CaMV and insertion into the pCanG expression vector, generating overexpression constructs of *p35S*::*DnFCAγ* and *p35S*::*DnFCAβ*, respectively. Then, these constructs were transformed into *Agrobacterium tumefaciens* EHA105, which was followed by introduction into *Arabidopsis* wild-type plants (in the *Col*-0 background) to create OX lines or into the *fca*-*1* mutant (in the L*er* background) to create HB lines using the floral dip method [43,44]. Phenotypic analysis was performed for homozygote lines at 22 ± 1 °C under a long-day (16 h light/8 h dark) or short-day (8 h light/16 h dark) photoperiod. The flowering time is defined as the time to the first flower opening after culturing under light [45]. The number of rosette leaves was counted at bolting.

### 2.4. Generation of Transgenic D. nobile

Before the transformation procedures, *Agrobacterium tumefaciens* EHA105 recombinants containing *p35S::DnFCAγ* or *p35S::DnFCAβ* constructs were recovered on YEB medium [46] with 50 mg/L kanamycin and 60 mg/L rifampin. Then, a monoclone of the EHA105 recombinant was picked and amplified in YEB liquid with kanamycin and rifampin until the OD_600_ of the culture reached close to 0.8. This culture was secondarily propagated on a large scale until the OD_600_ reached 0.6. Fresh cultures were centrifuged at 5000 rpm for 5 min. The precipitates were collected and suspended in 2 mL MS liquid [47], followed by the addition of fresh MS liquid and 200 μL acetosyringone to adjust the OD_600_ to a final value close to 0.6. Then, these EHA105 recombinants were co-cultured at 28 °C for 30–60 min with young seedlings generated by tissue culture or AXBs collected from adult stalks before the mixtures were vacuumed 1–2 times (10 min/time). After removal of the residual liquid from the surface, all plant materials were placed onto the MS medium (containing 100 μmol/L acetosyringone), followed by culturing in the dark for 3–5 days. As a control (CK), sterile deionized ultra-pure water was transformed independently in parallel.

### 2.5. Transcript Quantitation in Response to Temperature Treatment

The temperature treatments were performed as described in Section 2.1, followed by the collection of AXBs or leaves from individual plants at indicated time points. The materials were collected from 3–5 plants and mixed together before total RNA extraction as described previously [42]. Following this, 1 μg total RNA was used in reverse transcription using MMLV reverse transcriptase (TOYOBO, Japan), and 0.5 μL cDNA products were used as a template in the qPCR analysis using THUNDERBIRD SYBR qPCR Mix (TOYOBO, Japan) in a final volume of 20 μL. The gene-specific primers for each tested gene are listed in Table S1. The primer pair qDnUBQ-F and qDnUBQ-R was used to amplify the *DnUBQ* gene (coding for ubiquitin), which served as the endogenous control for normalization. The qPCR reactions were run in a Bio-Rad C1000 Thermo-cycler. Triplicate samples were analyzed independently. The data were collected and analyzed using Bio-Rad real-time PCR detection systems and software.

### 2.6. Data Analysis and Statistical Tests

All data collected in this study were analyzed and plotted in graphs using IBM SPSS Statistics 29.0 or GraphPad Prism 9. The built-in methods of one-way ANOVA, correlation, or t tests were used dependently and are described in Section 3 and the figure captions.

## 3. Results

### 3.1. DnFCA Produces γ and β Isoforms by Alternative 3′ Polyadenylation

**Figure 1:**
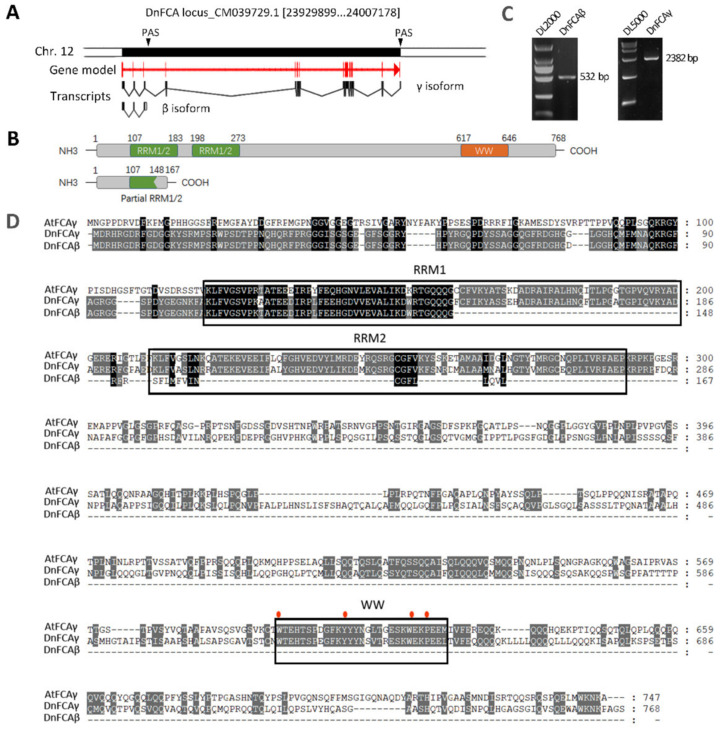
**Isolation of *DnFCA* isoforms**. (**A**) Processing models of the *DnFCAγ* and *β* isoforms. A diagrammatic drawing of the chromosome region locating the FKF09_017173 locus is shown on the top of the panel. The coding region is represented by the long black rectangle, and flanking regions are shown by non-colorful blocks. Two 3′ polyadenylation sites (PAS) that were predicted to add the 3′ poly (**A**) tail are shown by downward arrowheads. The gene model of the DnFCA (FLOWERING OCNTROL LOCUS C in *D. nobile*) protein is shown in red, while the processing models are shown in black. Arrows indicate the 5′→3′ direction of the gene. Exons are indicated using blocks; introns are shown as lines. The processing models were drawn using the “Exon–Intron Graphic Maker” online tool (accessed on 18 December 2022, http://www.wormweb.org/exonintron) based on the alignment analysis of the isoforms with the genomic sequence. (**B**) Models for deduced amino acid sequences of DnFCAγ and DnFCAβ. Conserved domains are colored in green for the RRM domains and in orange for the WW motif, as indicated. Numbers indicate the locations of the starting and ending sites of each domain or the first and the last residues of the peptide. (**C**) PCR verification of the *γ* and *β* isoforms in axillary buds (AXBs). Total RNA was extracted from AXBs of adult stalks, and RT-PCRs were performed using the primer pairs DnFCA-γ-F/DnFCA-γ-R and DnFCA-β-F/DnFCA-β-R (Table S1) to amplify the complete coding regions of the *DnFCAγ* and *β* isoforms, respectively. (**D**) Alignment analysis based on the amino acid sequences of DnFCAγ, DnFCAβ, and *Arabidopsis* FCAγ. The amino acid residues are shaded in black for 100% identities in all tested sequences and in grey for >60% identities. Conserved RRM domains and the WW motif are framed in black. The conserved amino acid residues within the WW motif are marked by red solid ovals.

In a preliminary experiment, a total of 15 transcript isoforms were detected by PacBio sequencing for the KFK09_017173 locus of the *D. nobile* genome [48], which was predicted to encode the FCA protein. Among these isoforms, at least two were produced through 3′ APA (Figure S1), a post-transcriptional processing that was also found in *Arabidopsis* to produce *FCA* transcript isoforms with different effects on flowering regulation [26]. Additionally, we also found that the *DnFCA* transcript was enriched in AXBs during vernalization at 10 °C/15 °C (night/day) [40]. These initial results led to a hypothesis that *DnFCA* might play roles in AXBs and is likely associated with the flowering nature of this species. To address this question and to further clarify the functional divergences between different 3′ APA isoforms, we first attempted to isolate the 3′ APA transcripts from the AXBs using the 3′RACE method in the current study. Consequently, only two isoforms of various lengths were identified (Figure 1C). BLASTN against the reference genome of *D. nobile* (GCA_022539455.1) revealed that both of them were products of the KFK09_017173 locus based on the addition of 3′ poly(A) tails at various PAS sites (Figure 1A,B), which is similar to what was reported for the production of *Arabidopsis FCA* isoforms [26]. The longer isoform was 3′ polyadenylated at the distal canonical PAS site downstream of the last exon, while the shorter one was produced using the proximal PAS located about 10 bp downstream of the “AAUAAA” element within the third intron (Figure 1A). The full-length ORF of the longer isoform codes for a peptide containing 768 aa (amino acids), which contains two conserved RNA recognition motif (RRM) domains and a WW motif (Figure 1D). BLASTX analysis indicated that this peptide is similar to *Arabidopsis* FCAγ and was subsequently named DnFCAγ. The shorter one was later named *DnFCAβ* due to the appending pattern of the 3′ poly(A) tail, which is similar to that of the *AtFCAβ* isoform [26]. The protein coding potential was then computed for this small isoform using CPC2 (accessed on 5 February 2023, http://cpc2.gao-lab.org/). Interestingly, the coding potential reached 0.989955 (Figure S2), suggesting that the *DnFCAβ* isoform may be a protein-coding sequence. *DnFCAβ* contains a short ORF (504 bp) and codes for a 167 aa peptide in prediction that does not contain any full-length RRM domains or a WW motif (Figure 1D). These results suggest that the *DnFCAγ* and *β* isoforms have different functions.

### 3.2. Expression of the DnFCAγ and β Isoforms

**Figure 2:**
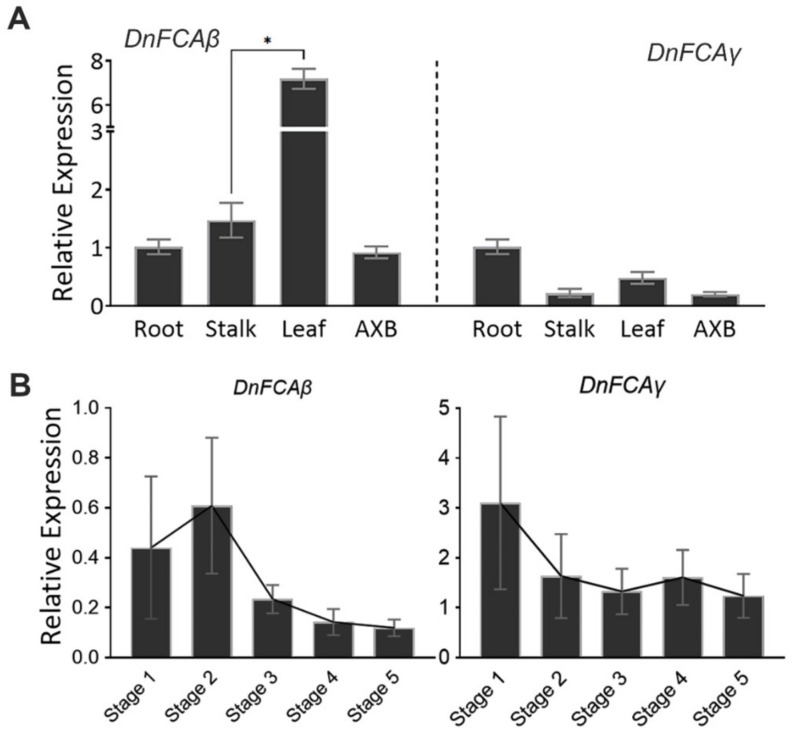
**Spatio-temporal accumulation of the *DnFCA* isoforms**. (**A**) Accumulation of the *DnFCAγ* and *DnFCAβ* isoforms in the tested plant organs. Total RNA was extracted from axillary buds (AXB), leaves (Leaf), newly emerged young roots (Root), and the upper part of 3-year-old adult stalks (Stalk) for RT-qPCR assays in triplicate. The height of each column indicates the mean expression level in indicated organs relative to that in roots (mean expression = 1). The expression of the *DnUBQ* gene was used as an endogenous control to normalize the expression of the target genes. Error bars show the SEM values. One-way ANOVA and Tukey’s multiple comparisons were performed for each gene to identify the significant differences between organs. Only those samples with significant differences are indicated by * *p* < 0.05. (**B**) Accumulation of the *DnFCAγ* and *DnFCAβ* isoforms in floral AXBs during development from Stage 1 to Stage 5. AXBs of 3-year-old adult stalks in vernalized plants were collected as described previously [41] and mixed for every development stage. A mixture of randomly collected AXBs from adult and non-adult stalks was used as the common control, and its expression level was set to 1 (not shown). Total RNA was extracted from each sample, and RT-qPCR reactions were conducted in triplicate. The expression of each gene was normalized by the expression of the *DnUBQ* gene (coding for ubiquitin). The column height indicates the mean expression level relative to that of the common control. One-way ANOVA and Dunnett’s multiple comparisons were performed to identify the differences between Stage 1 and every other stage. However, no significant differences were detected. Error bars represent the SEM values.

To clarify the spatio-temporal expressions of the *DnFCA* isoforms, reverse transcription qPCR assays were conducted using nonvernalized samples from 3-year-old adult stalks as starting materials. Compared with that in the roots, the abundance of the *DnFCAγ* isoform in the leaves, AXBs, and the upper part of the stalks was relatively low, with more than a twofold decrease (Figure 2A). The pattern was different for that of the *DnFCAβ* isoform, which was highest in the leaves, but similar in the buds, stalks, and roots (Figure 2A). After vernalization, the enrichments of the *DnFCAγ* and *β* isoforms were altered along with the development of floral AXBs. The relative abundance of *DnFCAγ* was consistently higher than that of *DnFCAβ* at each development stage. Nevertheless, the overall change trends of the *γ* and *β* isoforms were similar. Both of them peaked at Stage 1 and 2, the stages that the AXBs are undifferentiated or are undergoing the initiation of FM [3,40], followed by a decrease after Stage 3 (Figure 2B). To explore the co-expression relationship with *DnAPL1*, Pearson’s correlation coefficients were calculated based on the time-course dynamics during the development of the floral AXBs, which showed that the r value was −0.4288 for *DnFCAγ* and −0.5609 for *DnFCAβ*, with a *p*-value >0.05.

### 3.3. DnFCAγ and β Isoforms Regulate Flowering in Arabidopsis

**Figure 3:**
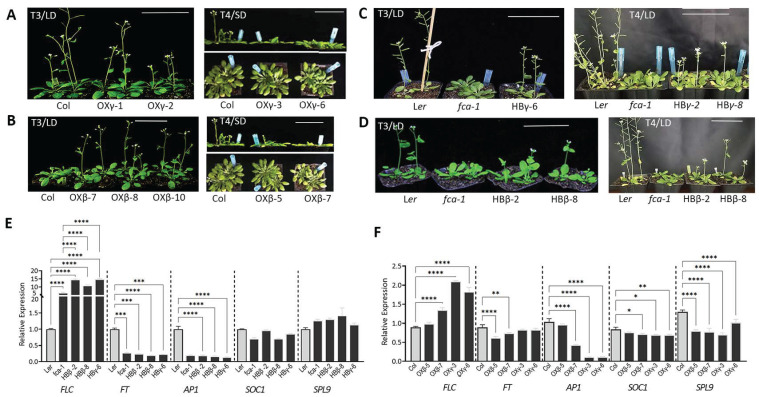
**Overexpression of dnfcaγ and dnfcaβ in arabidopsis.** (**A**–**D**) Flowering phenotypes of *DnFCAγ* (**A**,**C**) and *DnFCAβ* (**B**,**D**) transgenic *Arabidopsis*. The photographs of the transgenic plants in the T3/T4 generation growing under long-day (LD) or short-day (SD) photoperiods were taken when obvious differences were observed. Overexpression lines (OX) were created by introducing the indicated isoform into wild-type *Arabidopsis* in a Columbia (Col) background, while the complementation lines (HB) were created by introducing the given isoform into the *fca*-*1* mutant in a Landsberg *erecta* (L*er*) background. Bar length = 5 cm. (**E**,**F**) Expression of selected genes in *DnFCAγ* or *DnFCAβ* transgenic *Arabidopsis* lines. All plants growing under a long-day photoperiod were used. At least three 15-day-old seedlings were included in each sample for RNA extraction. RT-qPCR was conducted, and expression levels relative to the common control were calculated. The expression of each gene in L*er* wild-type seedlings was used as a common control and was set to 1. The *AtACTIN7* gene was used as an endogenous control for normalization. Expressions in complementation lines (HB) are shown in (**E**), and those in overexpression lines (OX) are shown in (**F**). Error bars indicate SEM values. One-way ANOVA and Dunnett’s multiple comparison were performed to identify the differences between the HB lines and the L*er* wild-type or *fca*-*1* mutant (**E**) or between OX lines and the Col wild-type (**F**). Pairs with significant differences are shown with * *p* < 0.05, ** *p* < 0.01, *** *p* < 0.001 and **** *p* < 0.0001.

We then determined whether these isoforms were involved in flowering regulation. Overexpression lines (OX) and complementation lines (HB) were created by introducing these two isoforms into *Arabidopsis* wild-type or the *fca*-*1* mutant. The phenotypic analysis of the HB lines indicated that both *DnFCAγ* and *DnFCAβ* were able to partially rescue the late flowering phenotype of the *fca*-*1* mutant (Figure 3C–E). However, although most of the *DnFCAγ*-OX lines in the T3 generation had a reduced number of rosette leaves than the wild-type plants under the long-day photoperiod (Table S2), the time taken to the opening of the first flower was extended in more than 50% of the transgenic lines (Table S2 and Figure 3A). When the T4 generation of the *DnFCAγ*-OX lines was analyzed, both parameters were increased accordingly in comparison to the wild-type (Table S2 and Figure 3A), and this phenotype was maintained in T5 and the later generations. A qPCR assay indicated that *DnFCAγ* and *DnFCAβ* were highly expressed in the corresponding OX or HB lines (Figure S3A,B). The abundance of *Arabidopsis AtFCAγ* was not altered, but *AtFCAβ* was highly accumulated in the OX lines (Figure S3C,D), similar to the observations in *AtFCAγ*-overexpressing lines [26]. Since *AtFCAβ* does not affect flowering in *Arabidopsis* [26], these results indicated that the phenotypes observed in the transgenic lines were caused by the overexpression of the exogenous *DnFCA* isoforms. In the *DnFCAγ*-OX lines, the expression of *AtFT* remained unchanged, but that of *AtSOC1*, *AtAP1,* and *AtSPL9* was significantly downregulated (Figure 3F). However, the expression of the flower suppressor, *AtFLC*, significantly increased (Figure 3F). Taken together, these results suggest that the *DnFCAγ* isoform tends to be a negative regulator of flowering, mostly by prolonging growth duration after bolting.

The *DnFCAβ*-OX lines were different. All of the T3 generation plants tested had a reduced number of rosette leaves and a shortened time to flowering (Table S3 and Figure 3B). However, even though the numbers of rosette leaves in the T4 generation were not different from those of the wild-type plants, the flowering time was uniformly delayed (Table S3 and Figure 3B), which also occurred in the later generations. The gene expression of the flowering regulator was then tested. As expected, *AtFT*, *AtSOC1*, *AtAP1*, and *AtSPL9* were suppressed in *DnFCAβ*-OX lines, with a *p*-value <0.05 in some samples (Figure 3F). However, At*FLC* was slightly, but significantly, upregulated in only one sample (Figure 3F).

### 3.4. Overexpression of the DnFCA Isoform in D. nobile

**Figure 4:**
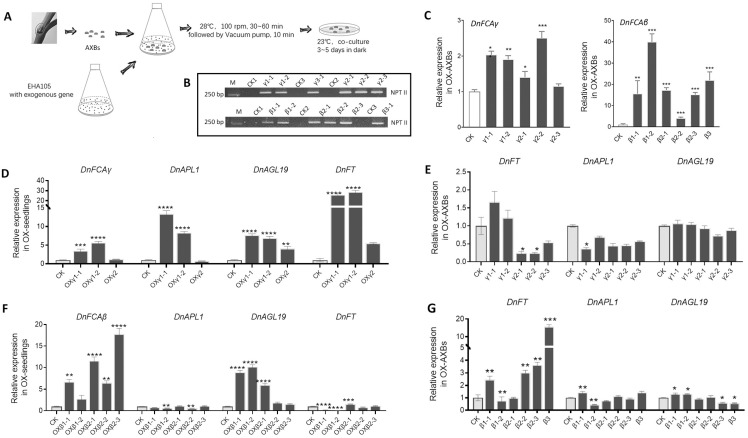
**Creation of transgenic AXBs or seedlings of *D. nobile***. (**A**) Schematic diagram of the work flow for creating transgenic *D. nobile* AXBs. To create transgenic AXBs, fresh AXBs from adult stalks were used. To create transgenic seedlings, young seedlings generated by tissue culture with three leaves were used. Expression constructs and detailed procedures are described in Section 2. (**B**) Expression verification of the *NPTII* gene in the suspected transgenic AXBs. The *NPTII* gene (coding for Neomycin phosphotransferase II protein) was used as the transgenic marker and was fused with the 35S promoter of CaMV to insert into the expression vector that harbors *p35S*::*DnFCAγ* or *p35S*::*DnFCAβ*, which was introduced into AXBs or seedlings. It was expected that the *NPTII* gene would be co-expressed with the *DnFCAγ* or *β* isoform and would indicate successful transformation and gene expression in the suspected transgenic materials. Total RNA extracted from the suspected materials was reverse-transcribed. Semi-quantitative PCR was then conducted, and the expression of the *NPTII* gene in transgenic AXBs and the control samples (CK1–3) was compared by the brightness of the bands on the agarose gel. M, DNA marker. (**C**) Overexpression verification for *DnFCA* isoforms in transgenic AXBs. Suspected transgenic AXBs and the control sample (CK) were collected for RNA extraction. Approximately 10–20 single AXB were mixed in each sample. RT-qPCR was conducted, and the expression levels of *DnFCAγ* (left) and *DnFCAβ* (right) were calculated, as described in Section 2. *DnUBQ* was used as an endogenous control to normalize the expression of the target genes. The expression level of the given gene in the control sample (CK) was set to 1, and the height of each column indicates the expression level relative to CK. One-way ANOVA and Dunnett’s multiple comparison were performed, and the transgenic samples that are significantly different from CK are indicated by * *p* < 0.05, ** *p* < 0.01, and *** *p* < 0.001. (**D**,**F**) Expression of *DnFT*, *DnAPL1*, and *DnAGL19* in *DnFCAγ* (**D** ) or *DnFCAβ* (**F**) transgenic seedlings. Overexpression verification of the *DnFCAγ* or *β* isoform and expression detection of the selected genes were performed using the same analysis methods as those in (**C**). *, **, ***, and **** indicate *p* < 0.05, <0.01, <0.001, and <0.0001, respectively. (**E**,**G**) Expression of *DnFT*, *DnAPL1*, and *DnAGL19* in *DnFCAγ* (**E**) or *DnFCAβ* (**G**) transgenic AXBs. The transgenic materials are same as those mentioned in (**C**). RNA extraction, RT-qPCR, and data analysis are the same as those in (**C**). *, **, and *** indicate *p* < 0.05, <0.01, and <0.001, respectively.

The differential phenotypes and gene expressions in transgenic *Arabidopsis* suggest that *DnFCAγ* and *DnFCAβ* may follow different pathways to regulate flowering. In order to further verify this in *D. nobile*, we created transgenic AXBs that transiently overexpressed the *γ* or *β* isoform (Figure 4A). RT-qPCR was used to pick out the transgenic AXB samples with higher expressions of the target *DnFCA* isoform and the co-introduced gene, *HPTII* (coding for Neomycin phosphotransferase II protein), in comparison with those in the control (CK). Consequently, we obtained five AXB samples overexpressing *DnFCAγ* and six overexpressing *DnFCAβ* (Figure 4B,C). As expected, *DnAPL1* was downregulated in *DnFCAγ*-overexpressing AXBs, while *DnAGL19*, a flowering promoter similar to *AtSOC1* [42], was not changed (Figure 4E). *DnFT* was downregulated in three of the five samples (Figure 4E). These results suggest that the overexpression of *DnFCAγ* could suppress the accumulation of the *DnAPL1* transcript in axillary buds. Comparatively, the overexpression of the *DnFCAβ* isoform in the AXBs led to the activation of *DnFT*, while the changes in *DnAGL19* and *DnAPL1* were not uniform across the tested samples (Figure 4G). Together, these results suggest that DnFCAγ may suppress *DnAPL1* expression in AXBs, while DnFCAβ might have positive effects on the expression of *DnFT*.

However, although the expression of *DnFT* was detected in the AXBs, similar to a previous report, it was suggested that the DnFT protein would be produced in the leaves rather than in the buds [49]. We then determined whether the expression of *DnFT* could be activated by the *DnFCA* isoforms in leaves. The *DnFCAγ* or *DnFCAβ* isoform was introduced into young seedlings of *D. nobile* (of which the leaves accounted for the most mass), generating transgenic materials overexpressing a given isoform (Figure 4D,F). In *DnFCAγ*-overexpressing seedlings, the expressions of *DnFT*, *DnAGL19*, and *DnAPL1* were significantly upregulated (Figure 4D). In comparison, only *DnAGL19* was uniformly activated in the *DnFCAβ*-overexpressing seedlings, while *DnAPL1* and *DnFT* were slightly suppressed in some samples (Figure 4F). Based on these results, it is likely that both the DnFCAγ and DnFCAβ proteins can serve as activators of *DnAGL19* in the leaves, whereas *DnFT* might be activated by DnFCAγ only.

### 3.5. Temperature-Induced Accumulation of DnFCA Isoforms

**Figure 5:**
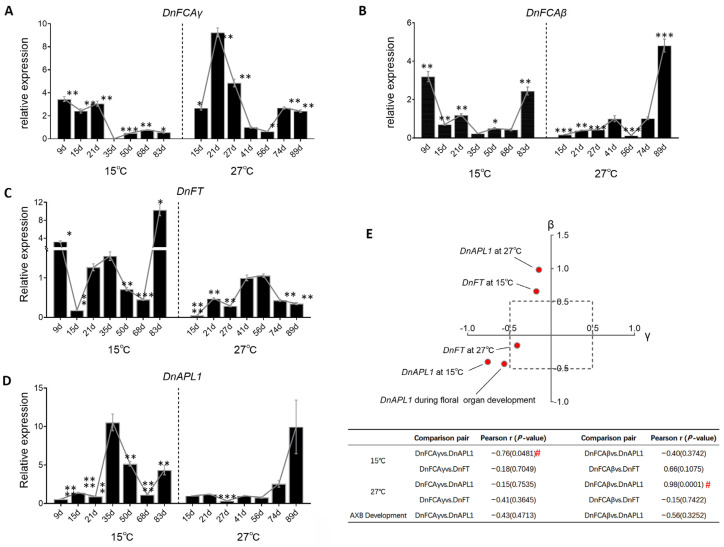
**Gene expression in AXBs in response to low or high temperatures**. (**A**–**D**) Time-course expressions of *DnFCAγ* (**A**), *DnFCAβ* (**B**), *DnFT* (**C**), and *DnAPL1* (**D**) in temperature-treated AXBs are shown. Temperature treatments were performed in culture chambers at 27 °C or 15 °C. Stage 1 AXBs were collected from 3-year-old adult stalks after 27 °C treatment at each time point. For treatment at 15 °C, AXBs were collected randomly from 3-year-old adult stalks. All AXBs collected before 50 d were at Stage 1, while after 50 d, the Stage 2 AXBs increased as the time extended. RNA was extracted from samples at each time point and used in RT-qPCR. The expression of each gene at 41 d at 27 °C was used as a common control and was set to 1. The expression at each time point was calibrated by this common control. One-way ANOVA and Dunnett’s multiple comparisons were performed to compare every other time point with the 35 d point at 15 °C or with the 41 d point at 27 °C; *p*-values were computed for each comparison. Time points with significant differences are marked by *, **, ***, and **** for *p* < 0.05, <0.01, <0.001, and <0.0001, respectively. (**E**) Co-expression relationship between *DnFCA* isoforms and *DnFT* or *DnAPL1* in AXBs. The co-expression relationship is reflected by Pearson’s correlation coefficients (r) and the corresponding two-tailed *p*-values, which were computed by comparing the time-course dynamics of each *DnFCA* isoform to those of *DnFT* or *DnAPL1*. The generated data are shown in the table below the plot, with red “#” indicating *p* < 0.05. Visually, the correlations are shown by red dots in the two-dimensional coordinate system. The location of each dot is determined by the r values of the indicated gene to *DnFCAγ* (X) and that to *DnFCAβ* (Y) and represents its co-expression tendency to these isoforms under the indicated condition. The closer a dot (a given gene) is to the upper part of the *Y*-axis, the stronger the positive co-expression with the *DnFCAβ* isoform. Similarly, the closer to the right of the *X*-axis, the stronger the positive co-expression with *DnFCAγ*.

If the regulation described above is true, it can be expected that co-expression should be observed between the *DnFCA* isoforms and the tested genes in naturally growing plants. The time-course accumulation of transcripts under high (27 °C) and low (15 °C) temperatures was then detected by RT-qPCR, and the Pearson correlation coefficients between the *DnFCA* isoforms and genes of interest were calculated. During the treatment at 15 °C, the *DnAPL1* transcript accumulated in the AXBs at a very low level before 35 d, which was followed by a dramatic increase (Figure 5D). This pattern is compatible with the initiation and growth of the petal and sepal at Stage 2/3, as reported previously [3,40]. Under treatment at 27 °C, the *DnAPL1* transcript was maintained at a very low level for a long time, although it increased after 89 days (Figure 5D). In contrast, the sum of the two *DnFCA* isoforms in the 15 °C-treated AXBs gradually decreased with time to the lowest value at 35 d and then slightly increased. The change in the *β* isoform was similar while that of the *γ* isoform was constantly higher before 35 d and decreased significantly thereafter (Figure 5A,B). The Pearson correlation coefficient (r) for *DnAPL1* accumulation was −0.7584 for the *γ* isoform with a *p*-value of <0.05 (Figure 5E), in agreement with the inhibitory effect of *DnFCAγ* on *DnAPL1* (Figure 4E). Under 27 °C, the *γ* isoform accumulated significantly at the earlier stages of treatment application (Figure 5A). Again, the change trend was negatively correlated with that of *DnAPL1* (Figure 5A,D). However, the yield of the *DnFCAγ* isoform decreased to some extent when the high temperatures continued (Figure 5A), resulting in a low r value for the overall change between *DnFCAγ* and *DnAPL1* (Figure 5E). Interestingly, the change in the *DnFCAβ* abundance was tightly correlated to that of *DnAPL1* (r = 0.9826, *p* < 0.0001) (Figure 5E). Since the overexpression of *DnFCAβ* did not exhibit an obvious regulatory effect on *DnAPL1* (Figure 4G), it is likely that there were other factors that could be attributed to the regulation of *DnAPL1* in this situation. Taken together, these results indicate that the suppression of *DnAPL1* expression by a high level of the *DnFCAγ* isoform is functional in low-temperature-treated AXBs, and likely also in high-temperature-treated ones.

**Figure 6:**
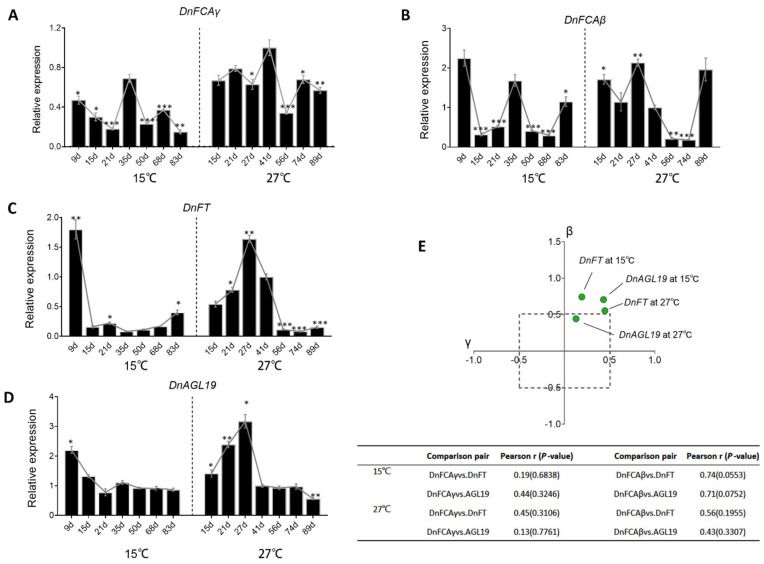
**Gene expression in leaves in response to low and high temperature.** (**A**–**D**) Time-course expressions of *DnFCAγ* (**A**), *DnFCAβ* (**B**), *DnFT* (**C**), and *DnAGL19* (**D**) in temperature-treated leaves. Temperature treatments were performed in culture chambers at 27 °C or 15 °C. Leaves were collected from 3-year-old adult stalks at indicated time points. RNA was extracted from each sample and was used in RT-qPCR. The expression of each gene at 27 °C at 41 d was used as a common control and was set to 1. The expression at each time point was calibrated by that in this common control. One-way ANOVA and Dunnett’s multiple comparisons were performed to compare every other time point to the 35 d point at 15 °C or to the 41 d point at 27 °C; *p*-values were computed for each comparison. Time points with a significant difference are marked by *, **, *** for *p* < 0.05, <0.01, <0.001, and <0.0001, respectively. (**E**) Co-expression relationship between *DnFCA* isoforms and *DnFT* or *DnAGL19* in leaves. The co-expression relationship is reflected by Pearson’s correlation coefficients (r) and the corresponding two-tailed *p*-values, which are computed by comparing the time-course dynamics of each *DnFCA* isoform to those of *DnFT* or *DnAPL1*. The generated data are shown in the table below the plot. Visually, the correlations are shown by green dots in the two-dimensional coordinate system. The location of each dot is determined by the r value of the indicated gene to *DnFCAγ* (X) and *DnFCAβ* (Y) and represents its co-expression tendency to these isoforms under the indicated condition. The closer a dot (a given gene) is to the upper part of the *Y*-axis, the stronger the positive co-expression with the *DnFCAβ* isoform. Similarly, the closer to the right side of the *X*-axis, the stronger the positive co-expression with *DnFCAγ*.

Since *DnFT* was activated by the overexpression of *DnFCAβ* in transgenic AXBs, we then addressed the hypothesis that *DnFT* would be upregulated in the natural AXBs with the accumulation of the *DnFCAβ* isoform. The expression of *DnFT* was then tested in temperature-treated AXBs. However, the results indicated that the expression of *DnFT* was very low in all tested AXB samples, with a peak only after long-term treatment at 15 °C (Figure 5C). The correlation coefficients were low for both the *DnFCAγ* and *β* isoform (Figure 5E), suggesting that *DnFT* would not be co-expressed with these two *DnFCA* isoforms in AXBs. We then determined whether *DnFT* could be activated in the leaves, as suggested by observations in transgenic seedlings (Figure 4D). The RT-qPCR analysis of the temperature-treated leaves revealed that *DnFT* was transiently activated after treatment at 15 °C for 9 days and was then dramatically decreased to a very low level (Figure 6C). However, at 27 °C, it was maintained at a high level for a long time, followed by a significant decrease after 50 days (Figure 6C). The accumulation pattern of the *DnFCAγ* isoform in these leaves was very different, being nearly steady with slight fluctuations at 15 °C and constantly high at 27 °C (Figure 6A). The co-expression relationship between *DnFCAγ* with *DnFT* in the leaves was positive and moderate at 27 °C, but weak at 15 °C (r = 0.1934 at 15 *°C*, 0.4484 at 27 °C) (Figure 6A,E). These data suggest that the activated effect of *DnFCAγ* accumulation on *DnFT* expression was weak in the leaves. The yield of the *DnFCAβ* isoform, on the other hand, fluctuated but had a decreasing trend at 15 °C. At 27 °C, this isoform accumulated highly only at the earlier stages (Figure 6B). Unexpectedly, the correlation coefficient of the *β* isoform to *DnFT* in the leaves was high, reaching 0.7435 at 15 °C and 0.5549 at 27 °C, with a *p*-value near 0.05 (Figure 6E), suggesting a positive but weak co-expression. This result is in disagreement with the fuzzy negative regulation of *DnFCAβ* on *DnFT* observed in transgenic seedlings (Figure 4F). Based on the discrepancy between the results from the transgenic experiments, we suggest that neither of the *DnFCA* isoforms can dramatically affect the expression of *DnFT* in leaves in response to temperature changes.

We considered the possibility that there may be a third factor that accounts for DnFCA’s regulation of *DnFT* (Figure 4D). *DnAGL19* was previously hypothesized to act upstream of *FT* in transgenic *Arabidopsis* [42]. Its expression was then detected in the temperature-treated leaves of *D. nobile*. As expected, the expression pattern of *DnAGL19* followed that of *DnFT* (Figure 6D), with a correlation coefficient up to 0.89 at 15 °C and 0.82 at 27 °C (*p*-value = 0.007 and 0.023, respectively). Additionally, *DnAGL19* was also positively co-expressed strongly with the *DnFCAβ* isoform but weakly with *DnFCAγ* (Figure 6E). Taken together, these findings support the notion that both *DnFCA* isoforms positively regulate *DnAGL19*, with the *β* isoform serving as the lead.

## 4. Discussion

It is well known that in *D. nobile* and other nobile-type *Dendrobium* species, exposure to low temperatures (vernalization) is required for AXBs to be released from dormancy, whereas constant exposure to high ambient temperatures will result in a failure to flower [1,2,3,40,50]. Generally, a low-temperature period of 30–40 days (but can range from 20 to 50 days) is sufficient for the formation of Stage 1 floral AXBs, which do not have any morphological changes visible to the naked eye, but the inflorescence meristems (IM) are initiated [3,40,41]. Consistent with this, the *D. nobile* orthologs of *Arabidopsis CONSTANS* (*CO)*, *AGL24*, *AGL19*, *FCA*, *FVE*, and *VIP4* are activated, while the indicator for floral meristem (FM) determination, *DnAPL1*, is still suppressed [40,41]. Extending the exposure to low temperatures leads to a further transition to Stage 2, which is accompanied by the activation of *DnAPL1* (Figure 5D). These findings agree with the morphological observations described in [3].

There are two loci on the *D. nobile* genome that are predicted to code for putative FCA proteins [48]. In the current study, we confirm that one of these, namely, the KFK09_017173 locus, can generate functional products for flowering regulation. *DnFCAγ* and *DnFCAβ* are two isoforms of this locus that are produced by 3′APA on pre-mRNA at different PAS and can be isolated from floral AXBs (Figure 1A,B). The 3′ APA mode to generate these two isoforms is conserved across species, which was found previously not only in *Arabidopsis* but also in rice [26,27]. The *γ* isoform, which is formed by adding a 3′ poly(A) tail at the canonical PAS, codes for a peptide with highly similar amino acid sequences across species. The *β* isoform, however, varies among different species at the 3′ terminus, containing species-specific sequences transcribed from a part of the large intron (Figure 1A) [26,27]. It thus can be expected that functional divergence would exist across species for the *β* isoforms due to their distinct sequences and the architectures of the predicted protein. In our present study, both the γ and β isoforms from *D. nobile* were found to be able to, but only partially, rescue the late flowering phenotype of the *fca*-*1* mutant (Figure 3), indicating that they have some biological and molecular functions similar to those of the AtFCAγ protein in *Arabidopsis* [26]. Nevertheless, functional differences were also observed, such as the regulation of *DnAPL1* and *DnAGL19* discussed below.

### 4.1. The DnFCAγ Protein Suppresses the Expression of DnAPL1 in AXBs

Phase transition requires the reprogramming of meristematic identities [51]. The MADS-box transcription factor AP1 in *Arabidopsis* serves as a hub that mediates the switch from floral induction to flower formation by promoting FM identity as well as the specification of sepals and petals [13,14]. The activators for *AP1* have been extensively explored [16,18], whereas information regarding its repressors is limited. There are some clues indicating that the FCA protein may act as a negative regulator of flower development. For example, the overexpression of *OsFCA* can induce the curling of leaves, the failure of flower opening, and non-seed setting in *Arabidopsis* [27]. The *Arabidopsis fca*-*1* mutant has an increased number of floral nodes and co-inflorescences [20], also suggesting that *Arabidopsis* FCAγ may serve as a repressor of floral meristem (FM) determination and axillary SAM initiation. Our findings in this current study provide advanced support for this idea. The *DnFCAγ* isoform does not predominantly contribute to floral transition in *Arabidopsis* but is able to delay the opening of the first flower (Figure 3 and Table S2). Furthermore, the expression of *Arabidopsis AP1* was significantly downregulated under *DnFCAγ* overexpression (Figure 3). Such a suppressive effect was further confirmed by *DnFCAγ*-overexpressing *D. nobile* AXBs, in which *DnAPL1*, the *AP1* ortholog in *D. nobile*, was suppressed (Figure 4). Although *DnAPL1* was also suppressed in *DnFCAβ*-overexpressing AXBs or seedlings, the effects were weak and not uniform across the tested samples and can be ignored (Figure 4). Together, these findings suggest a suppressive model of DnFCAγ–DnAPL1, which is predominantly functional in AXBs to regulate floral bud development in a timely manner.

The negative co-expression between *DnFCAγ* and *DnAPL1* provides strong support for this model to work during low-temperature-induced phase transition and floral bud development (Figure 2, Figure 5 and Figure 6). Stage 1 floral AXBs that accumulate *DnFCAγ* after sufficient spells of low temperatures (e.g., 15 °C for 20–50 days) can be attributed to low levels of the *DnAPL1* transcript. Such suppression may be obligatory, by which the premature initiation and development of floral buds can be avoided at this time. One interesting question is whether this suppression is related to the delay of flower blooming, which typically occurs after a long winter. Based on our results, this suggestion is partially supported. The expression of *DnAPL1* can be released from the suppression by DnFCAγ after 35 days at low temperature, which provides opportunities for growth transition from Stage 1 to Stage 2/3 (Figure 5A,D) to achieve the development of the sepals and petals [3]. This transition is slow and fluctuates under long periods of low temperatures and is related to a delay in flowering. It remains unclear whether this DnFCAγ–DnAPL1 pathway is functional in terms of the early flowering induced by the well-timed application of warm temperatures. The temperature treatments used in our study were limited, so an answer to this question must be sought in future research. On the other hand, the negative correlation between *DnFCAγ* and *DnAPL1* also suggests that the non-flowering phenotype induced by long-term constant high temperatures may be a consequence of the continual suppression of *DnAPL1* by the high accumulation of *DnFCAγ* under such a condition (Figure 5A,D). It was noted that an increase in *DnAPL1* was observed after a very long treatment time at 27 °C (Figure 5D). It is unclear whether this activation is related to leaf development in emerging offshoots [41].

VRN2 in wheat can serve as a negative input in the leaves to suppress *VRN1*, an *AP1*-like gene, which can result in the transcriptional inhibition of *FT1* and the prevention of flowering under suboptimal conditions [24,25]. We present another possible pathway in our study for the suppression of *AP1*-like genes in undifferentiated floral buds. It is unclear whether this pathway is conserved in orchids or other non-orchid species. However, it was noted that *DOAP1*, an *AP1*-like gene in other *Dendrobium* species, was previously reported as an A-class floral organ identity gene and has some functions similar to those of *DnAPL1* of *D. nobile* [35,41]. The promotion of early flowering by this gene was found to be accompanied by a significant decrease in the number of floral buds in transgenic orchid plants [35], leading to the hypothesis that it is the premature accumulation of DOAP1 that blocks the transition from IM to FM in shoot apexes. It is interesting to test whether the *FCAγ* homolog in this species is involved in the suppression of the DOAP1-associated premature termination. The regulation of DnFCAγ to *DnAPL1* in *D. nobile* may be indirect, while the components bridging them were not explored in the present study. In *Arabidopsis*, LEAFY (LFY) serves as a pioneer transcription factor, binding the nucleosome-rich regions of *AtAP1* chromatin (promoter and the first intron) to activate *AtAP1* expression in seedling leaves [52]. It also acts as a direct regulator of *AtAP1* at shoot tips at the early stage of flower development [14,16]. In the perennial plant *Arabis alpine*, the *LFY* ortholog, *AaLFY*, and its suppressor AaTFL1 were suggested to account for the age-dependent flowering and the repeated flowering phenotype [53,54]. *D. nobile* has similar flowering phenotypes to those of *Arabis alpine* [53]. In this context, it is interesting to determine whether the *LFY* homolog can be used as the intermediate to transmit the regulation of DnFCAγ to *DnAPL1*. Unfortunately, the *LFY* homolog has not been functionally characterized due to the lack of reliable sequences. More in-depth studies are required to unravel the relationship between these genes. On the other hand, it was also noted that the expressional correlation between the *β* isoform and *DnAPL1* was also high under high temperatures (Figure 5). This result disagrees with the unchanged expression of *DnAPL1* in *DnFCAβ*-overexpressing AXBs (Figure 4). A reasonable explanation is that *DnFCAβ* may serve as a transcript balancer rather than a protein-coding transcript. Similar to *AtFCAβ* in *Arabidopsis* [26], *DnFCAβ* is used to ensure the relative high yield of the *DnFCAγ* isoform under this condition by dragging down the usage of the proximal PAS. This suggestion should be verified in further studies.

### 4.2. The DnFCAβ Protein Induces the Expression of DnAGL19 in Leaves

The findings in this study also put forward the hypothesis that the *DnFCA* gene serves as an activator of *DnAGL19* expression in leaves (Figure 4D,F and Figure 6). Such activation was more dominant for the *DnFCAβ* isoform , particularly, under low temperatures (Figure 6E). It is likely that the induction of *DnFT* expression in leaves may be one of the possible consequences of the activation of DnFCAβ-DnAGAL19, as *DnAGL19* was reported to promote flowering in transgenic *Arabidopsis* via the “HOS1-FT” module [42] and was also found to collaborate with *DnFT* in the leaves regardless of the temperature to which the plants were exposed (Figure 5 and Figure 6). Taken together, it appears that the *DnFCAβ* isoform may act in the leaves to contribute to the activation of DnFT, which is mediated by *DnAGL19*. As a mobile signal, the DnFT protein will be transported to axillary shoot apexes to promote floral transition and IM determination [49]. Notably, the activation and transport of DnFT can occur very early and rapidly after low-temperature application (Figure 6), which agrees with the claims in previous studies that floral transition can be achieved shortly after about 2 weeks of vernalization in some *Dendrobium* plants [1,3,40]. This pathway might be functional under high temperatures, which is weakly based on the correlation measurements (Figure 6E). Nevertheless, the effect of such an induction of *DnFT* may be futile, as the concurrent accumulation of *DnFCAγ* in AXBs at the same time can inhibit *DnAPL1* and block the further development of floral buds.

## 5. Conclusions and Perspectives

**Figure 7:**
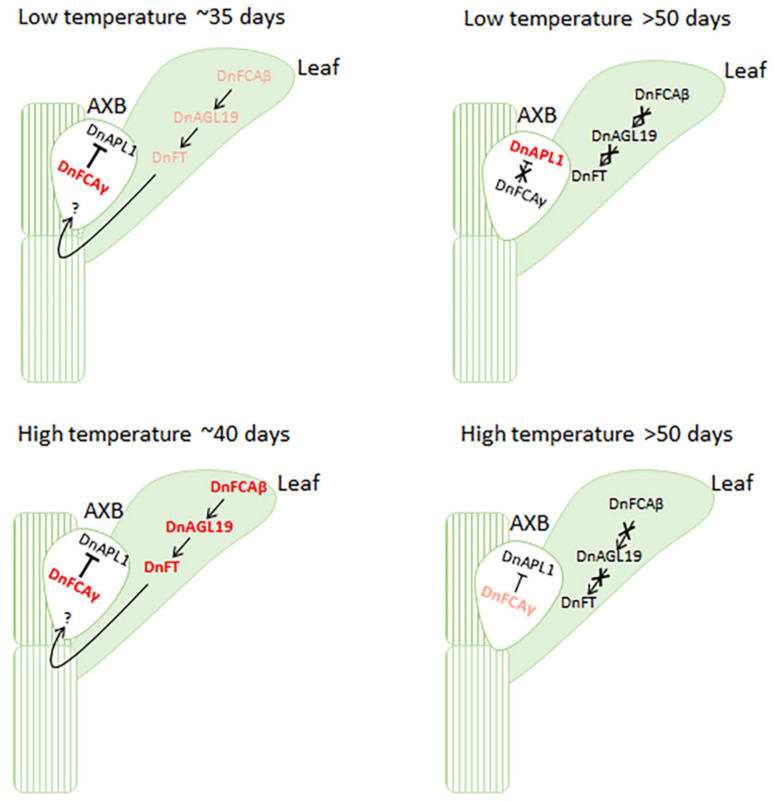
**The “DnFCAγ–DnAPL1” and “DnFCAβ–DnAGL19” regulation pathways for temperature-related flowering in *D. nobile***. The “DnFCAγ–DnAPL1” pathway in AXBs and the “DnFCAβ–DnAGL19” pathway in leaves proposed to regulate flowering in *D. nobile* are shown. In brief, a relatively short period of low temperatures can efficiently induce a high level of accumulation of the *DnFCAβ* transcript in leaves, which allows the transient activation of *DnAGL19* to elevate the expression of *DnFT* within a very short period. The resultant DnFT proteins are transported into AXBs to promote floral transition, ensuring differentiation of IM. At the same time, the high-level accumulation of the *DnFCAγ* transcript in AXBs leads to the suppression of *DnAPL1*, preventing the premature development of floral organs. A long period of low temperatures can lead to a decrease in *DnFCAγ* in AXBs, releasing *DnAPL1* from suppression to promote the development of inflorescence and floral organs. Non-flowering under a constant high temperature may be due to the high yield of *DnFCAγ* in AXBs, even if *DnFT* is activated through the “DnFCAβ–DnAGL19” pathway in leaves. The abundance of a transcript of a given gene or isoform is shown by the color of the font. The red font indicates a high level of transcription, the light red font indicates moderate activation of transcription, and the black font indicates a low level of transcription. “→” reflects “activation”, “┫” indicates “suppression”, and “×” indicates the failure of a given regulation. The thickness of the arrow lines indicates the regulation strength.

In summary, we provide a reasonable regulation model underlying the flowering induced by low temperatures and underlying the non-flowering phenotype under a constant high temperature, in which two isoforms from the *DnFCA* locus cooperate to regulate floral transition and flower development (Figure 7). DnFCAγ serves as a suppressor of *DnAPL1* and negatively regulates floral organ development in AXBs. However, DnFCAβ may function as an activator of *DnAGL19* in the leaves and probably also of *DnFT* indirectly. When *D. nobile* plants are vernalized by low temperatures, *DnAGL19* is upregulated by the transient accumulation of the *DnFCAβ* isoform in leaves, which probably results in the activation of *DnFT* and the rapid transport of the florigen into AXBs, allowing the vegetative-to-reproductive phase transition. The high level of *DnFCAγ* at this time may suppress the expression of *DnAPL1*, probably, along with some other A-function genes, inhibiting the premature development of floral organs. This ensures the complete floral transition and maturation of IM and is of benefit to the subsequent floral organ determination and growth. The suppression of *DnAPL1* will disappear after the yield of the *DnFCAγ* isoform is downregulated by a long period of low temperature, allowing the differentiation and development of floral organs. The high-temperature-induced accumulation of *DnFCAβ* in the leaves also leads to a similar activation of *DnAGL19* in leaves and probably results in DnFT input into AXBs. However, the concurrent accumulation of *DnFCAγ* in AXBs, which will be maintained for a long time, disrupts the flowering process at the transition from IM to FM due to the inactivation of *DnAPL1*.

It should be noted that there are many questions that cannot be answered based on this model. For example, does this regulation network contribute to other flowering traits, such as floral reversion in spring, the outgrowth of offshoots under constant warm temperatures, and early flowering due to the well-timed application of warm treatment after vernalization? It is necessary to identify the intermediate components bridging the *DnFCA* isoforms to *DnAPL1* or *DnAGL19* and to discover the underlying molecular mechanisms. On the other hand, it would be very interesting to discover how the *DnFCAβ* isoform works molecularly. As this isoform is so small and the fact that it contains no known functional domains in prediction (Figure 1), there may be multiple possible modes of its action. For example, the *β* isoform could serve as non-coding regulatory RNA and be involved in transcription of the *DnFCA* gene or could act as a transcript balancer to adjust the yield of the *DnFCAγ* isoform. However, the high possibility of protein-coding potential for the *DnFCAβ* isoform suggests it would act in the protein form, probably serving as a competitor of the DnFCAγ protein or playing an independent role. Answers to these questions are beyond the scope of the present study and remain to be discovered in the future.

## Data Availability

The sequences of *DnFCAγ* and *DnFCAβ* have been deposited in Genbank under accession No. OQ148170 and OQ148171, respectively.

## Supplementary Materials

The following supporting information can be downloaded at https://www.mdpi.com/article/10.3390/biology12020331/s1. The file contains Figure S1: The isoforms of the KFK09_017173 locus of the *D. nobile* genome; Figure S2: Protein-coding potential of the *DnFCAβ* isoform; Figure S3: Expression verification of the *DnFCA* and *AtFCA* isoforms in the transgenic *Arabidopsis* lines; Table S1: Primers used in this study; Table S2: Flowering phenotypes of *DnFCAγ* transgenic *Arabidopsis*; and Table S3: Flowering phenotypes of *DnFCAβ* transgenic *Arabidopsis.*
