# Supplementary material for: DnFCA Isoforms Cooperatively Regulate Temperature-Related Flowering in Dendrobium nobile
Source: Biology (Basel). 2023 Feb 19;12(2):331. doi: 10.3390/biology12020331 (PMC9953494; doi:10.3390/biology12020331)
Supplement: Supplementary file 1 [file biology-12-00331-s001.zip › biology-2148028-supplementary.pdf]

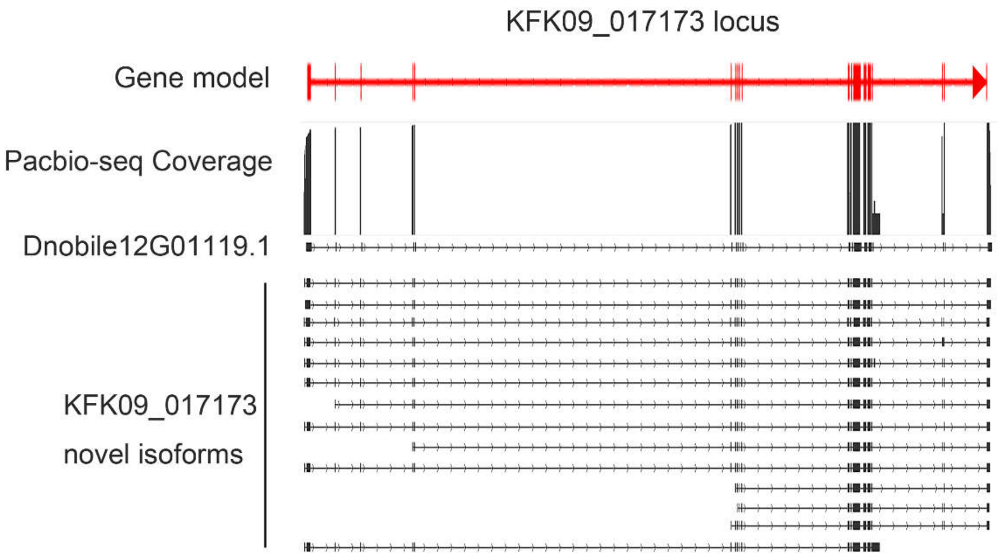

Figure S1. The isoforms of the KFK09_017173 locus of the *D. nobile* genome. KFK09_017173 contains coding region for a putative DnFCA protein. A total of 15 isoforms were identified for this locus based on data from PacBio sequencing of mixed AXBs (unpublished data). The Dnobile12G01119.1 is the representative transcript model for this locus and is also confirmed to express in AXBs by PacBio sequencing. Other 14 isoforms were predicted based on the alignment analysis of sequences against the reference genome of *D. nobile* (GCA_022539455.1). The arrowheads indicate the 5’ →3’ direction of the putative *DnFCA* gene or transcript isoform. Black blocks in the isoform models and red blocks in the gene model represent exons, black lines in the isoform models and red lines in the gene model represent introns. The coverage of PacBio sequences to the representative gene model are shown as black peaks.

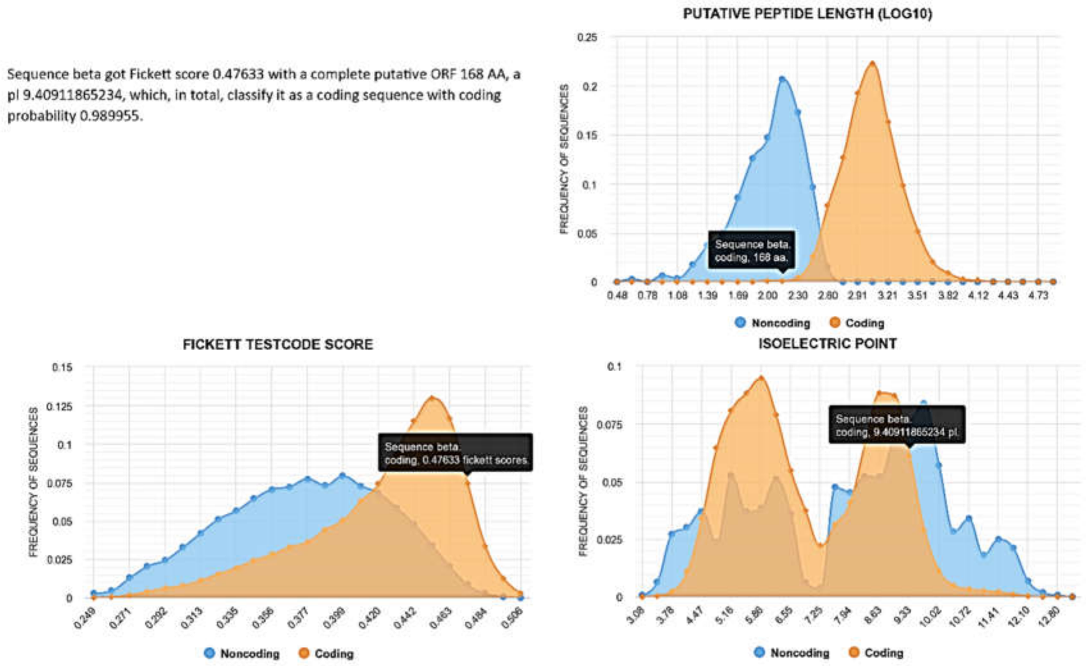

Figure S2. Protein coding potential of the *DnFCAβ* isoform. The potential of *DnFCAβ* isoform was analyzed using CPC2 at http://cpc2.gao-lab.org/ using default parameters. The peptide length and isoelectric point of *DnFCAβ* were computed, and the Fickett score was 0.4763. The *DnFCAβ* was classiafied as a protein-coding sequence with a probability up to 0.9899.

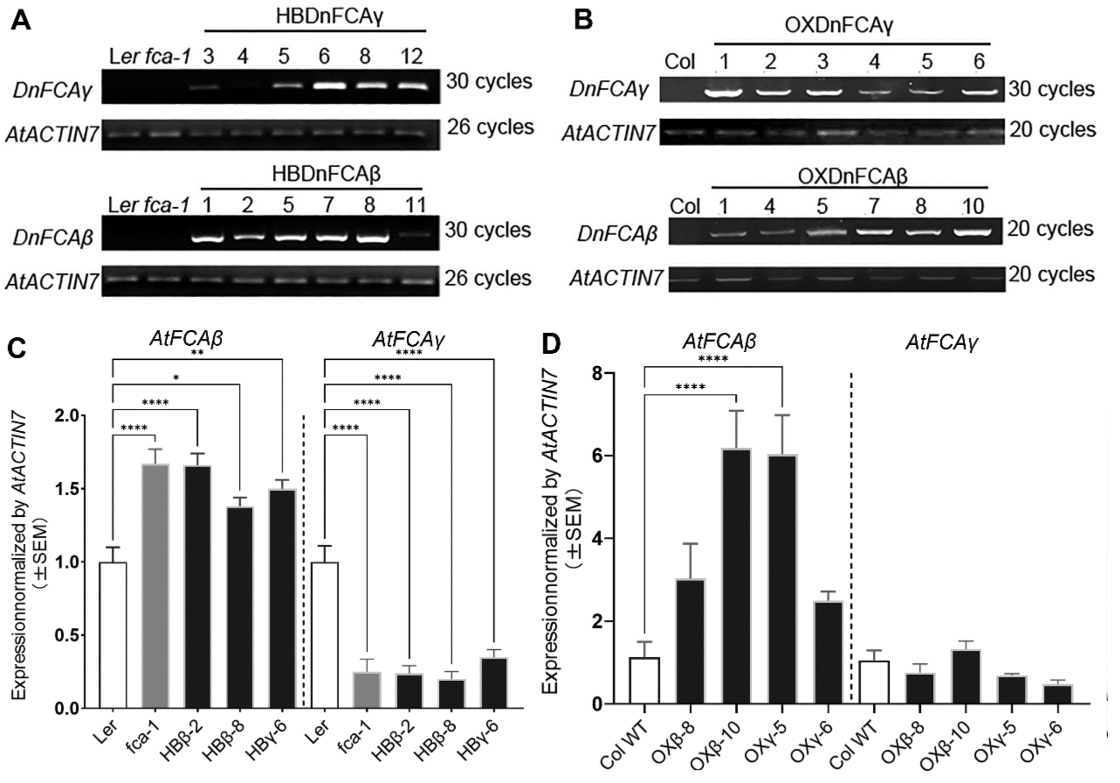

Figure S3. Expression verification of the *DnFCA* and *AtFCA* isoforms in the transgenic *Arabidopsis* lines. (A-B) Overexpression of *DnFCAγ* or *DnFCAβ* in OX **(B)** and HB **(A)** lines. 15-day-old seedlings of the six homozygous lines were collected and total RNA was extracted, followed by reverse transcription for 1 μg total RNA in a final volume of 20 μl. 1 μl resultant RT product was used for a semi-quantitative PCR reaction in a final volume of 20 μl. The gene specific primers corresponding to *DnFCAγ* or *DnFCAβ* isoform are listed in Table S1. The gene of *AtACTIN 7* served as the endogenous control for expression normalization. PCR reactions stopped after running for the indicated thermal cycles and 5 μl PCR products were tested by electrophoresis on 1% agarose gel. **(C-D)** Expression of *AtFCAγ* and *AtFCAβ* isoform in *DnFCA* OX **(D)** and HB **(C)** lines. Transgenic *Arabidopsis* with high expression of the exogenous genes of *DnFCAγ* or *DnFCAβ* were selected for this experiment. Total RNA was extracted from 15-day-old seedlings and 1 μg of the extractive products was reverse transcribed into cDNA in a 20 μl reaction. 0.5 μl cDNA was used a template in qPCR reaction with a reaction with final volume of 20 μl. qPCR reactions were run in Bio-Rad C1000 Thermo-cycler. The *AtACTIN7* was used as internal control for expression normalization. Expression of each tested gene in wild-type plants was set to 1 and used as the comparing control. One-way ANOVA was performed to identify the significant differences. All HB lines were compared with the L*er* wild type and the *fca-1* mutant. OX lines were compared with the Col wild type. The strength of significance is indicated by **p*<0.05, ***p*<0.01 or *****p*<0.0001.

Table S1. Primers used in this study.

primer	sequence (5'→3')
qDnFT-F	GCCAAGCCTAGGCATACATCGC
qDnFT-R	CAGTCTTGCATTCTTCTTCCGCC
qDnAGL19-F	CTCTGCTGTGCCTAAATGCT
qDnAGL19-R	AACTGTTGGGTTCTTCCTCC
qDnAPL1-F1	TCTTACTCGGGTGAATAGCAACAGC
qDnAPL1-R1	TAGCCGGATTTGATTAGCTCGCATT
realtimeDnFCA–β-F	TATGGTGAGGGAAACAAA
realtimeDnFCA–β-R	AAGAAAACCACAATTTATCACGA
realtimeDnFCA -γ-F	GCAACGGGACCTATTCAAG
realtimeDnFCA -γ-R	CCACGACTCTGTTTCATCTCA
qDnUBQ-F	AAAGGCTCATCTTTGCCGGT
qDnUBQ-R	CTCTTAGCCACCATCACCAAATTGC
anchor Oligo(dT)	ATTCTAGAGGCCGAGGCGGCCGACATG-d(T)$_{30}$VN
3' PCR Primer	ATTCTAGAGGCCGAGGCGGCCGACATG
Dn FCA-γ-F	GAGCAGATAACAGCGGAGC
Dn FCA-γ-R	CTCGTTAAAGATTCAAAAGTCGTAGC
Dn FCA-β-F	CAACCTCTCGAATACGATGGACAG
Dn FCA-β-R	GATCCCCAAGCCTCAAAG
AtcFLC_F236	GTCGCTCTTCTCGTCGTCTC
AtcFLC_R427	TCCCACAAGCTTGCTATCCAC
ATcSOC1-_F517	CTCTCAGTGCTTTGTGATGCTG
ATcSOC1-_R686	TTCAAATGCTGCATATTTTCTTCAG
AtcFT_F249	TTGGTGGAGAAGACCTCAGGA
AtcFT_R393	TCATTGCCAAAGGTTGTTCCAG
AtcAP1_F340	TGCTCTTGTTGTCTTCTCCCA
AtcAP1_R485	ACCAGTTTGTATTGACGTCGGA
qAtACT7-F2	AGATCACCGCTCTTGCACCT
qAtACT7-R2	ACTCACTCTTTGAAATCCACATCTGTT

Table S2. Flowering phenotypes of *DnFCAγ* transgenic *Arabidopsis*.

Line #	Generation	n	RL	Flowering time
**Long-day**				
Col	T3	12	8.54±0.72	33.56±2.46
OXγ-1	T3	12	8.23±0.86	33.48±2.72
OXγl-2	T3	12	8.11±0.83	31.79±2.65
Col	T3	12	7.79±0.51	29.10±1.85
OXγl-5	T3	12	8.42±1.14	31.69±2.88**
OXγ-6	T3	12	8.39±1.196	31.15±2.93
Col	T3	12	8.21±1.03	29.53±2.64
OXγ-3	T3	12	7.89±0.77	30.45±2.53
OXγl-4	T3	12	7.79±0.41	28.21±1.69
Col	T4	12	9.25±0.68	30.23±1.98*
OXγl-1	T4	12	8.64±0.79*	31.76±2.51*
OXγ-2	T4	12	8.72±0.67*	33.39±2.12**
OXγ-3	T4	12	8.9±0.73*	32.53±2.843*
OXγ-4	T4	12	8.75±0.68*	31.94±2.58*
Col	T4	8	5.875±0.35	29.63±1.06
OXγ-1	T4	12	5.08±0.29**	31.00±0.43**
OXγ-2	T4	12	5.58±0.51	30.67±1.07*
L*er*	T3	11	5.82±0.75	30.45±0.82
fca-1	T3	12	7.17±1.03**	32.75±0.75**
HBγ-6	T3	12	6.83±0.72**	32.83±0.83**
L*er*	T4	11	7.18±0.60	29.09±1.58
fca-1	T4	12	9.83±0.72^{a}	37.92±3.50^{a}
HBγ-6	T4	18	10.33±0.76^{a}	39.61±3.52^{a}
HBγ-8	T4	16	10.75±0.74^{ab}	41.38±1.82^{ab}
**Short-day**				
Col	T4	16	14.25±1.183	50.13±1.09
OXγ-3	T4	16	16.00±1.51*	51.63±1.20*
OXγ-6	T4	16	16.25±1.39*	51.82±0.98*
L*er*	T4	16	8.81±0.83	39.50±2.50
fca-1	T4	16	24.44±2.19^{a}	NF§
HBγ-6	T4	16	14.38±2.156^{ab}	49.67±2.60^{a}

RL, the number of rosette leaves at bloting; Flowering time, Days to the opening of the first flower. ***p*<0.001; * *p*<0.05; ^{a} p<0.05 in comparing with L*er*; ^{b} p<0.05 in comparing with *fca-1*; ND, no records; NF§, No flowers until the observed date.

Table S3. Flowering phenotypes of *DnFCAβ* transgenic *Arabidopsis*.

Line #	Generation	n	RL at bolting	Days to flowering
**Long-day**				
Col	T3	12	7.63±0.82	28.13±1.83
OXβ-5	T3	12	7.00±0.73*	26.46±1.48**
OXβ-7	T3	12	7.22±0.64	26.18±1.41**
Col	T3	12	8.83±0.96	30.31±1.90
OXβ-8	T3	12	7.78±1.33*	28.54±1.85**
OXβ-10	T3	12	8.19±0.47*	28.61±1.88*
Col	T3	12	7.46±0.59	29.06±2.42
OXβ-1	T3	12	7.53±0.57	29.87±2.49
OXβ-4	T3	12	7.47±0.61	29.43±2.54
Col	T4	24	6.13±0.68	30.71±1.33
OXβ-5	T4	12	5.58±0.67*	32.00±1.04*
OXβ-1	T4	12	6.00±0.60	31.67±1.30*
OXβ-4	T4	12	5.83±0.72	32.83±0.58**
OXβ-7	T4	12	6.55±0.90**	31.45±0.69**
OXβ-8	T4	12	6.25±0.89**	31.08±0.45**
L*er*	T3	12	5.58±0.79	31.42±1.00
fca-1	T3	10	7.42±0.51[a]	33.50±0.85[a]
HBβ-2	T3	12	6.25±0.97[ab]	32.50±0.67[ab]
HBβ-1	T3	12	6.75±0.75[a]	33.08±0.67[ab]
HBβ-8	T3	12	6.17±0.94[b]	32.25±0.75
HBβ-11	T3	12	7.08±0.79[a]	33.18±0.60[a]
L*er*	T4	12	ND	30.17±1.03
fca-1	T4	12	ND	40.25±1.75**
HBβ-2	T4	12	ND	40.06±3.34**
HBβ-8	T4	12	ND	38.94±2.93**
**Short-day**				
COL	T4	16	12±0.9661	38.75±1.5706
β-7-3	T4	16	9.74±0.86**	35.19±1.36**
β-8-7	T4	16	9.38±0.50**	34.82±1.45**
β-10-3	T4	16	9.69±0.48**	35.56±1.24**
L*er*	T4	16	8.79±0.80	39.50±1.51
fca-1	T4	16	24.40±2.16[a]	NF[§]
HBβ-2	T4	16	17.38±2.66[ab]	54.80±3.26[a]
HBβ-8	T4	16	17.80±3.27[ab]	55.00±3.36[a]
HBβ-6	T4	16	14.40±2.09[ab]	49.66±2.56[a]

RL, the number of rosette leaves at bloting; Flowering time, Days to the opening of the first flower

**p<0.001; * p<0.05; [a] p<0.05 in comparing with Ler; [b] p<0.05 in comparing with fca-1

ND, no records; NF[§], No flowers until the observed date
